# IL-17A-producing γδ T cells promote liver pathology in acute murine schistosomiasis

**DOI:** 10.1186/s13071-020-04200-4

**Published:** 2020-07-01

**Authors:** Lei Sun, Wenci Gong, Yujuan Shen, Le Liang, Xiaofan Zhang, Teng Li, Tina Tuwen Chen, Yuan Hu, Jianping Cao

**Affiliations:** 1 Key Laboratory of Parasite and Vector Biology, Ministry of Health, Shanghai, 200025 China; 2 National Institute of Parasitic Diseases, Chinese Center for Disease Control and Prevention, Shanghai, 200025 China; 3 Chinese Center for Tropical Diseases Research, Shanghai, 200025 China; 4 WHO Collaborating Centre for Tropical Diseases, Shanghai, 200025 China; 5 National Center for International Research on Tropical Diseases, Ministry of Science and Technology, Shanghai, 200025 China

**Keywords:** γδ T cells, IL-17A, Granuloma, Fibrosis, Schistosomiasis

## Abstract

**Background:**

The main symptoms of schistosomiasis are granuloma and fibrosis, caused by *Schistosoma* eggs. Numerous types of cells and cytokines are involved in the progression of *Schistosoma* infection. As a class of innate immune cells, γδ T cells play critical roles in the early immune response. However, their role in modulating granuloma and fibrosis remains to be clarified.

**Methods:**

Liver fibrosis in wild-type (WT) mice and T cell receptor (TCR) δ knockout (KO) mice infected with *Schistosoma japonicum* was examined *via* Masson’s trichrome staining of collagen deposition and quantitative reverse transcriptase-PCR (RT-PCR) of fibrosis-related genes. Granuloma was detected by hematoxylin-eosin (H&E) staining and quantified. Flow cytometry was used for immune cell profiling and for detecting cytokine secretion. The abundance of the related cytokines was measured using quantitative RT-PCR.

**Results:**

The livers of *S. japonicum*-infected mice had significantly increased proportions of interleukin (IL)-17A producing γδ T cells and secreted IL-17A. Compared with the WT mice, TCR δ deficiency resulted in reduced pathological impairment and fibrosis in the liver and increased survival in infected mice. In addition, the profibrogenic effects of γδ T cells in infected mice were associated with enhanced CD11b^+^Gr-1^+^ cells, concurrent with increased expression of transforming growth factor (TGF)-β in the liver.

**Conclusions:**

In this mouse model of *Schistosoma* infection, γδ T cells may promote liver fibrosis by recruiting CD11b^+^Gr-1^+^ cells. These findings shed new light on the pathogenesis of liver pathology in murine schistosomiasis.
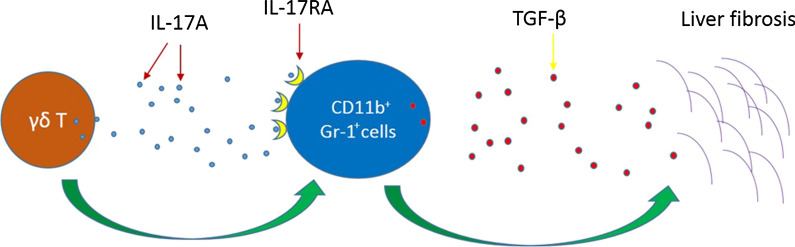

## Background

Schistosomiasis is a neglected tropical disease caused by the parasites of the genus *Schistosoma.* Infection is prevalent in over 74 countries, and nearly 200 million people are at risk [1–3]. Following infection, liver fibrosis is the major pathological manifestation of the disease. This is mainly due to the deposition of *Schistosoma* eggs in the host liver. The excretory secretory antigens produced by the eggs induce strong immune responses and inflammation, and trigger granuloma formation around the eggs, which in turn leads to the activation of hepatic stellate cells (HSCs) and consequent liver fibrosis [4].

The mechanisms of fibrosis have been studied extensively in the murine model of schistosomiasis. One major mechanism is the switching of the immune pattern from a predominant T helper cell 1 (Th1) response to a Th2-dominant response, which promotes granuloma and fibrosis formation by secreting cytokines such as interleukin (IL)-4, IL-5 and IL-13 [5–9]. As a member of the IL-17 family, IL-17A is involved in various biological processes by binding its cognate receptor, IL-17RA [10–13]. Accumulating evidence demonstrates that IL-17A is related to a variety of inflammatory and autoimmune diseases such as inflammatory bowel diseases (IBD) and multiple sclerosis [14–16]. Multiple studies have demonstrated that Th17 cells, natural killer T cells and γδ T cells produce IL-17A [17–19]. Th17 promotes hepatic granuloma formation by secreting IL-17A [20–22]. Moreover, the absence of IL-17A signaling has been implicated in alleviating liver fibrosis in murine schistosomiasis [23].

Multiple cell types, including macrophages, T follicular helper cells (Tfh), regulatory T cells (Treg), and dendritic cells (DC) are activated and involved in granuloma formation and in the process of fibrosis [24–28]. γδ T cells are a subgroup of T cells that express a distinct T cell receptor (TCR) consisting of a γ and a δ chain, which allows rapid response to antigens without the requirement of major histocompatibility complex (MHC) presentation [29, 30]. γδ T cells play pivotal roles in autoimmunity, inflammatory diseases, and tumor development [31–34]. They also have important regulatory functions [35]. Of note, they are an important regulator in liver diseases, including liver infection, non-alcoholic fatty liver disease, autoimmune hepatitis, and liver fibrosis [36]. Although a correlation between γδ T cell and neutrophil recruitment and liver fibrosis has been implicated [37], the exact mechanism underlying the regulatory role of γδ T cells in liver fibrosis remains elusive. In the present study, we aimed to characterize the role of γδ T cells in the early formation of granuloma and fibrosis in the livers of TCR δ knockout (KO) mice infected with *Schistosoma japonicum*. We provide evidence that deleting TCR δ markedly ameliorates liver fibrosis and improves liver function. We explored the mechanism by which γδ T cells promote liver pathology in murine schistosomiasis. Our findings indicate that γδ T cells may promote liver fibrosis by recruiting CD11b^+^Gr-1^+^ cells, which thereby furthers understanding of fibrosis formation and provides a strategy for the basis of anti-schistosomiasis therapy.

## Methods

### Mice, parasite and infection

Eight-week-old male C57BL/6 mice were purchased from Shanghai Jiesijie Animal Feeding Co. Ltd. (Shanghai, China). TCR δ KO mice on C57BL/6 background were purchased from Jackson Laboratory (Bar Harbor, USA) and bred in our facility. For the comparison experiment, wild-type (WT) and KO mice were bred under specific pathogen-free conditions. In the infection experiments, 31 WT mice and 31 KO mice were each infected percutaneously *via* the shaved skin of the abdomen, with 20 ± 2 *S. japonicum* cercariae obtained from the National Institute of Parasitic Diseases, Chinese Center for Disease Control and Prevention (Shanghai, China). Fifteen WT mice and 15 TCR δ KO mice were randomly divided into three groups, and were sacrificed for pathological observation at 0, 4 and 6 weeks post-infection, respectively. Sixteen WT mice and 16 KO mice were used for the survival time observation, and the survival rates in each group were evaluated.

### Liver pathology and measurement of aspartate aminotransferase/alanine aminotransferase (AST/ALT) levels

Fresh liver samples were fixed in 10% neutral buffered formalin, then processed with routine paraffin-embedding procedures. The paraffin-embedded sections (5 μm) were dewaxed and stained with hematoxylin-eosin (H&E) or Masson’s trichrome for analyzing granuloma or fibrosis, respectively. Granulomas around single eggs were selected to assess the levels of granuloma and fibrosis. Each stained section was examined by optical microscopy under 100× magnification with identical settings. Thirty pictures were taken of granulomas around single eggs from three sections in each tissue. The areas featuring granuloma and fibrosis surrounding a single egg were estimated using ImageJ (NIH, Bethesda, MD, USA). Sera from individual infected mice were separated by centrifugation at 1000× *g* for 10 min. ALT and AST levels were detected by Adicon Clinical Laboratories, Inc. (Shanghai, China).

### Isolation of liver non-parenchymal cells and spleen cells

Single-cell suspension of hepatic leukocytes were prepared using a conventional method according to a previous report with some modifications [17]. Briefly, infected or control mice were perfused with phosphate-buffered saline (PBS) *via* the portal vein. The excised liver was minced, then pressed through a 70-μm cell strainer (BD Biosciences, San Jose, CA, USA). The total hepatic cells were then resuspended in 40% Percoll solution (GE Healthcare, Chicago, IL, USA), and centrifuged for 15 min at 750× *g*. Then, the leukocytes were resuspended in erythrocyte-lysing buffer. Lastly, the cells were washed and resuspended in RPMI 1640 medium. For the spleen, all cells were washed and resuspended in RPMI 1640 medium immediately after they had been pressed through a 70-μm cell strainer (BD Biosciences).

### Flow cytometry

To assess cytokine and biological marker expression on γδ T cells and CD11b^+^Gr-1^+^ cells, 2 × 10^6^ cells per 100 μl were incubated with the following fluorescence-labeled antibodies for 30 min at 4 °C; fluorescein isothiocyanate (FITC) labeled anti-CD3; phycoerythrin (PE)-Cy7-labeled anti-γδ TCR; allophycocyanin (APC)-labeled anti-Vγ1; PE-labeled anti-Vγ2; Brilliant Violet 421-labeled anti-CD11b; APC-labeled anti-Gr-1; and PE-labeled anti-IL17RA (BioLegend, San Diego, CA, USA). After surface marker staining, the cells were permeabilized with Fix/Perm buffer (BD Biosciences) for 15 min, then incubated with the following fluorescence-labeled antibodies: Brilliant Violet 421-labeled anti-IL17A; Brilliant Violet 421-labeled anti-interferon (IFN)-γ; and PE-labeled anti-transforming growth factor (TGF)-β. The staining panels were as follows: γδ T cell, FITC-labeled anti-CD3, PE-Cy7-labeled anti-γδ TCR, APC-labeled anti-Vγ1, PE-labeled anti-Vγ2, Brilliant Violet 421-labeled anti-IL17A/Brilliant Violet 421-labeled anti-IFN-γ; CD11b^+^Gr-1^+^ cells, Brilliant Violet 421-labeled anti-CD11b, APC-labeled anti-Gr-1, PE-labeled anti-TGF-β/PE-labeled anti-IL17RA.

All flow cytometry data were acquired on an LSRFortessa X-20 cell analyzer (BD Biosciences) and analyzed with FlowJo software (Tree Star, Ashland, OR, USA).

### Quantitative reverse transcriptase PCR (qRT-PCR)

The total RNA was extracted from liver tissue with TRIzol (Invitrogen, Carlsbad, CA, USA). For each sample, 2 μg of total RNA was reverse-transcribed using a complementary DNA (cDNA) reverse transcription kit (TaKaRa, Dalian, China). The comparative threshold cycle (2^−ΔΔCq^) was calculated to evaluate the relative mRNA expression, and glyceraldehyde-3-phosphate dehydrogenase (GAPDH) levels were used as a normalization control. The qRT-PCR primers used in this study are listed in Table 1.

**Table 1:** Primer sequences used in qRT-PCR analysis

Gene	Forward (5′–3′)	Reverse (5′–3′)
GADPH	GAGCCAAACGGGTCATCATCT	GAGGGGCCATCCACAGTCTT
TGF-β	GACCGCAACAACGCCATCTA	GGCGTATCAGTGGGGGTCAG
IL-13	TGAGCAACATCACACAAGACC	GGCCTTGCGGTTACAGAGG
IL-17A	GCTCCAGAAGGCCCTCAGA	CTTTCCCTCCGCATTGACA

### Statistical analysis

All quantitative data were reported as the mean and standard error of the mean (SE). All samples were compared using an unpaired Student’s t-test or one-way analysis of variance (ANOVA). The survival rate was analysed by using the Kaplan–Meier method, the difference between survival curves was tested for statistical significance using the log-rank test. *P* < 0.05 was considered to indicate statistical significance. GraphPad Prism 6 (GraphPad Software, San Diego, CA, USA) was used for all statistical analysis and graphs.

## Results

### The liver γδ T cells of infected mice had significantly increased IL-17A secretion

**Fig. 1:**
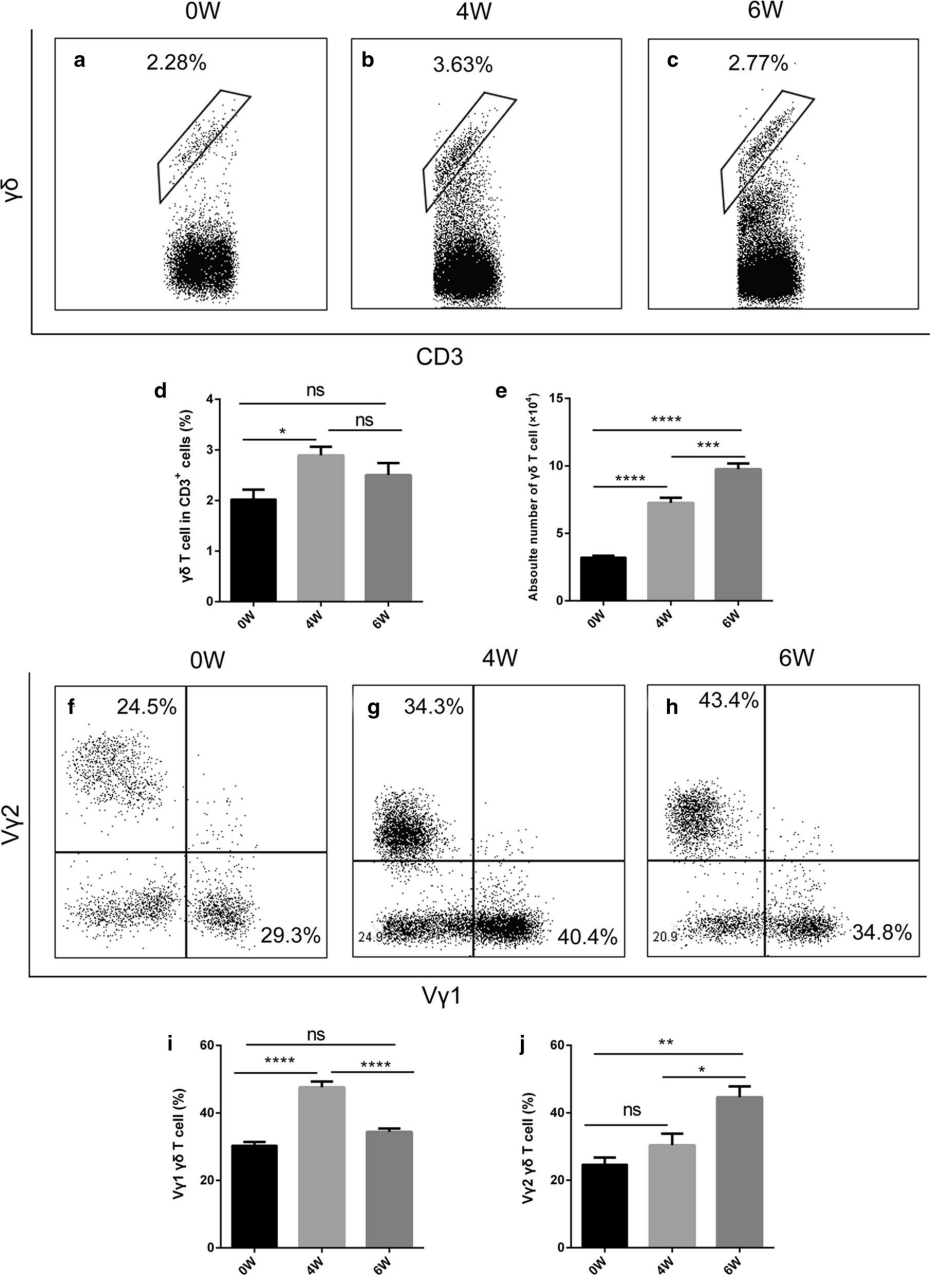
Percentage of γδ T cells and their subsets in *S. japonicum*-infected mouse liver. **a**-**c** Flow cytometry analysis of percentage of γδ T cells in total CD3^+^ cells from liver leukocytes, and the representative dot plots at 0 (**a**), 4 (**b**) and 6 (**c**) weeks post-infection. **d**, **e** Statistical analysis of the percentage (**d**) and absolute number (**e**) of γδ T cells in CD3^+^ cells from liver leukocytes. **f**-**h** Flow cytometry analysis of Vγ1 and Vγ2 subset percentages among γδ T cells in total CD3^+^ T cells from liver leukocytes at 0 (**f**), 4 (**g**) and 6 (**h**) weeks post-infection, and the representative dot plots. **i**-**j** Statistical analysis of the percentage of Vγ1 (**i**) and Vγ2 (**j**) cells in total CD3^+^ T cells from liver leukocytes. The differences at 0, 4 and 6 weeks were analyzed using a one-way analysis of variance (ANOVA), *n* = 5 per group, *P < 0.05, **P < 0.01, ***P < 0.001, ****P < 0.0001; ns, no significant difference

**Fig. 2:**
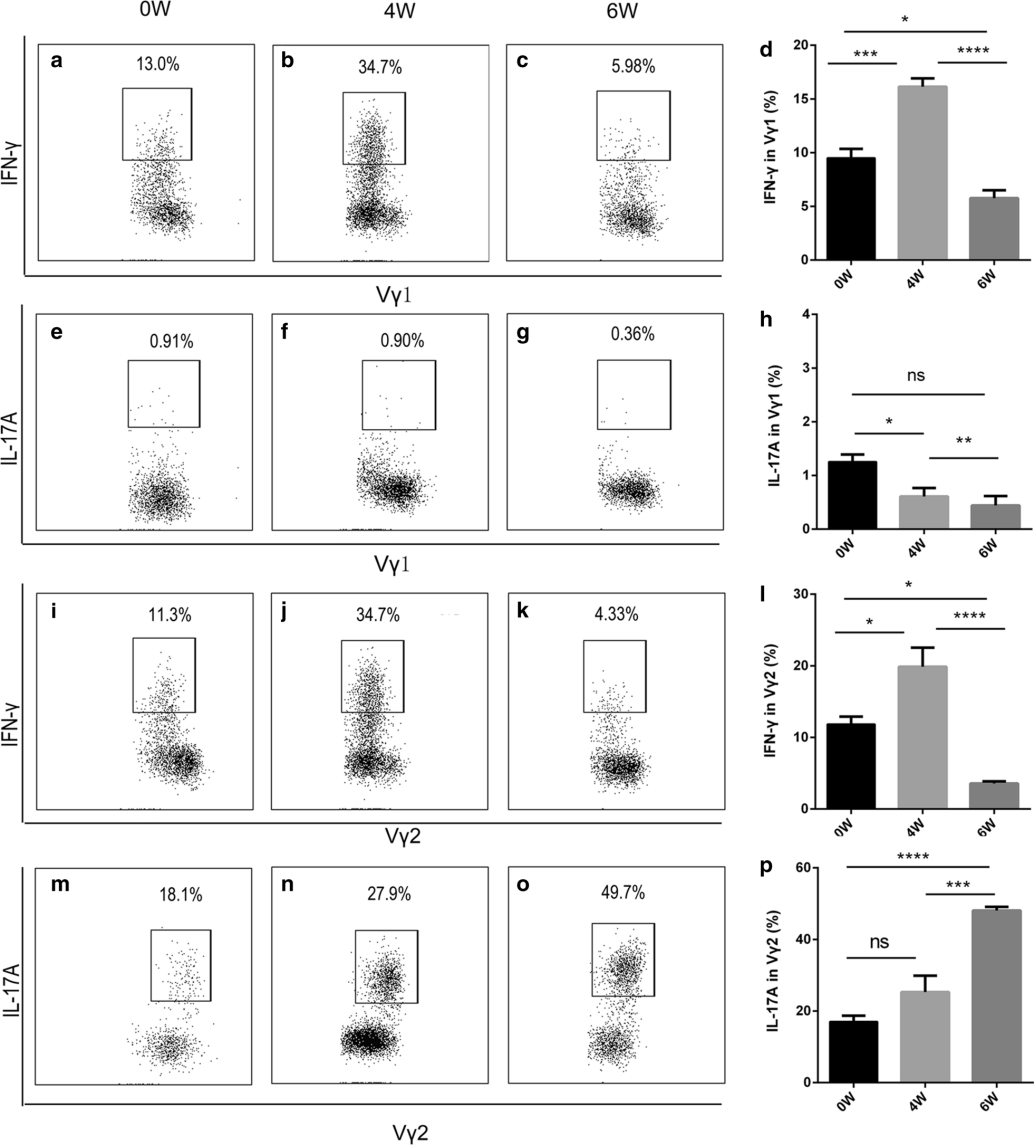
Vγ1 and Vγ2 cells secrete IFN-γ and IL-17A, respectively, in the liver. **a**-**d** Flow cytometric and statistical analyses of the percentage of IFN-γ from Vγ1 cells at 0 (**a**), 4 (**b**) and 6 (**c**) weeks post-infection. **e**-**h** Flow cytometric and statistical analyses of the percentage of IL-17A from Vγ1 cells at 0 (**e**), 4 (**f**) and 6 (**g**) weeks post-infection. **i**-**l** Flow cytometric and statistical analyses of the percentage of IFN-γ from Vγ2 cells at 0 (**i**), 4 (**j**) and 6 (**k**) weeks post-infection. **m**-**p** Flow cytometric and statistical analyses of the percentage of IL-17A from Vγ2 cells at 0 (**m**), 4 (**n**) and 6 (**o**) weeks post-infection. Liver leukocytes were stimulated with PMA (phorbol 12-myristate 13-acetate) +ionomycin for 4 h; intracellular IL-17A and IFN-γ staining were performed and representative dot plots are shown. The differences at 0, 4 and 6 weeks were analyzed using a one-way ANOVA, *n* = 5 per group, *P < 0.05, **P < 0.01, ***P < 0.001, ****P < 0.0001; ns, no significant difference

To profile the dynamic changes of the γδ T cells, we performed fluorescence-activated cell sorting and quantified the ratio of γδ T cells in the liver of *S. japonicum*-infected mice. Additional file 1: Figure S1 shows the gating strategy. The γδ T cells constituted approximately 2%, 3% and 2.8% of the total CD3^+^ T cells in the liver at 0, 4 and 6 weeks post-infection, respectively (Fig. 1a-c). During infection, the proportion of γδ T cells did not change greatly (*F*_(2, 12)_ = 4.657, *P* = 0.0318) (Fig. 1d). However, the absolute number of γδ T cells was approximately 4.0 × 10^4^, 7.5 × 10^4^ and 10.0 × 10^4^ at 0, 4 and 6 weeks post-infection, respectively, which was a significant increase (*F*_(2, 12)_ = 100.7, *P* < 0.0001) (Fig. 1e). Notably, γδ T cell subtype analysis revealed mainly two subtypes: Vγ1 and Vγ2 (Fig. 1f-h). The percentage of Vγ1 cells peaked at 4 weeks post-infection (*F*_(2, 12)_ = 49.46, *P* < 0.0001) (Fig. 1i), whereas that of the Vγ2 cells increased gradually post-infection (*F*_(2, 12)_ = 11.98, *P* = 0.0014) (Fig. 1j). We then detected the cytokines secreted by the two subtypes. Additional file 2: Figure S2a-b shows the isotype controls. Vγ1 cells mainly secreted IFN-γ (Fig. 2a-c) rather than IL-17A (*F*_(2, 12)_ = 7.481, *P* = 0.0078) (Fig. 2e-h), while Vγ2 cells secreted both IFN-γ (Fig. 2i-k) and IL-17A (Fig. 2m-o). IFN-γ from the Vγ1 cells increased at 4 weeks post-infection, and then decreased at 6 weeks post-infection (*F*_(2, 12)_ = 44.56, *P* < 0.0001) (Fig. 2d), as did IFN-γ from the Vγ2 cells (*F*_(2, 12)_ = 23.84, *P* < 0.0001) (Fig. 2l). However, IL-17A from the Vγ2 cells increased during infection (*F*_(2, 12)_ = 31.37, *P* < 0.0001) (Fig. 2p). These results suggest that γδ T cells may participate in the liver pathological process in mice infected with *S. japonicum.*

### Infected TCR δ KO mice had alleviated pathology and liver damage

**Fig. 3:**
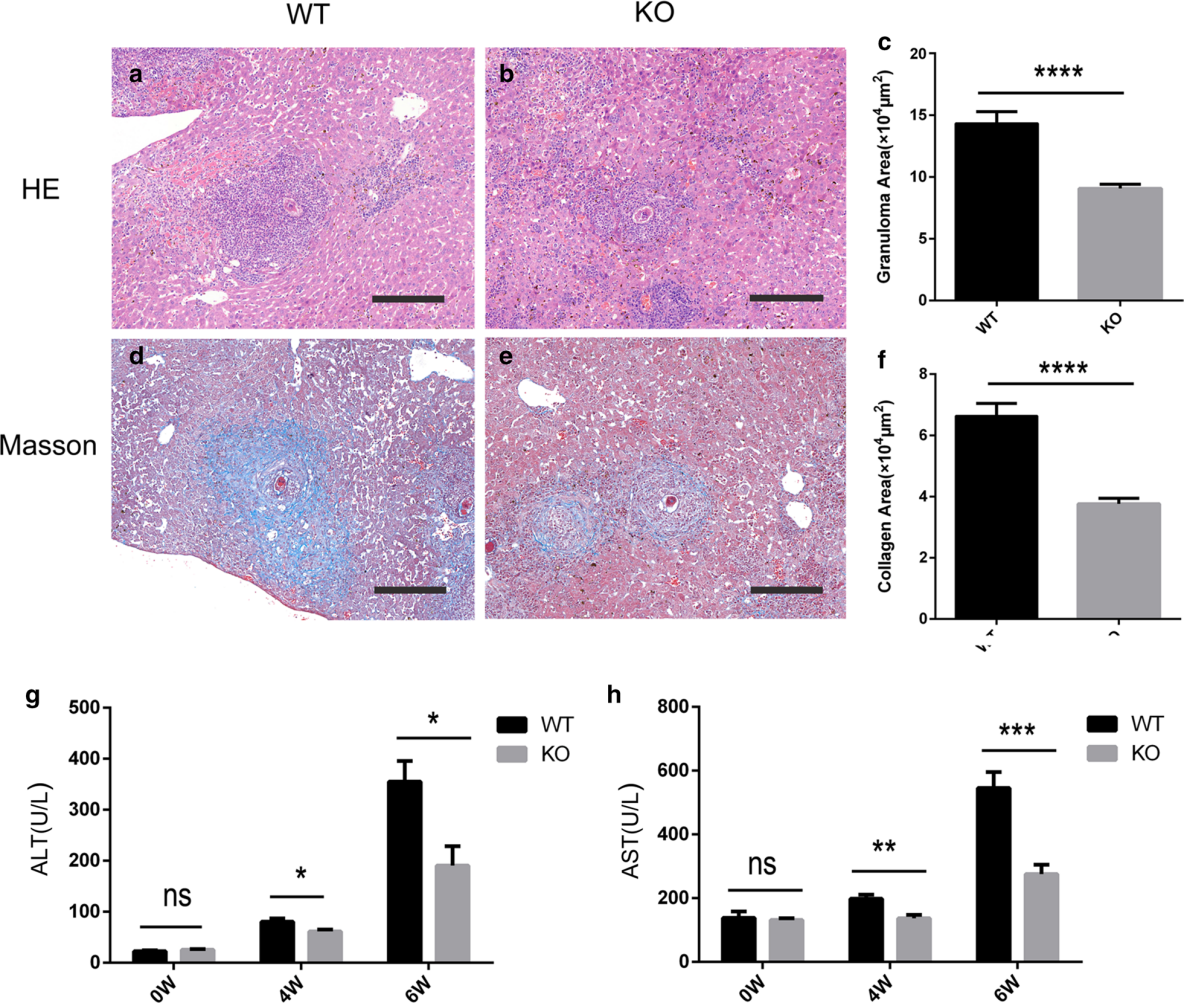
Decreased fibrosis and liver damage in *S. japonicum*-infected TCR δ KO mice. **a**, **b** H&E staining showing liver granuloma in WT (**a**) and TCR δ KO (**b**) mice at 6 weeks post-infection. **c** Statistical analysis of the granuloma area between the two groups. **d**, **e** Masson staining showing the liver fibrosis in WT (**d**) and KO (**e**) mice at 6 weeks post-infection. **f** Statistical analysis of the fibrosis area between the two groups. **g**, **h** Statistical analysis of serum ALT (**g**) and AST (**h**) in WT and KO mice at 0, 4 and 6 weeks post-infection. Differences were analyzed by using a Student’s t-test, *n* = 5 per group *P < 0.05, **P < 0.01, ***P < 0.001, ****P < 0.0001; ns, no significant difference

To evaluate whether γδ T cells were involved in the pathogenesis of *S. japonicum* infection in mice, WT and TCR δ KO mice were infected with *S. japonicum*, and were sacrificed at 6 weeks post-infection. Hepatic granuloma size was determined using H&E staining (Fig. 3a, b). The average granuloma area in the KO mouse livers was much less than that of the WT mice (*t*_(58)_ = 5.248, *P* < 0.0001) (Fig. 3c). Collagen deposition was detected using Masson’s trichrome staining (Fig. 3d, e), the KO mouse livers had significantly less severe fibrosis than that of the WT mice (*t*_(58)_ = 6.481, *P* < 0.0001) (Fig. 3f). We also determined liver function (ALT/AST) at 0, 4 and 6 weeks after infection. The KO mice showed improved liver function in comparison with the WT mice at 4 and 6 weeks post-infection (*t*_(8)_ = 2.690, *P* = 0.0275, *t*_(8)_ = 2.982, *P* = 0.0175; and *t*_(8)_ = 3.626, *P* = 0.0067, *t*_(8)_ = 4.691, *P* = 0.0016, respectively) (Fig. 3g, h). Further, we monitored the survival rates of the mice. From week 11 onwards, the KO mice had significantly higher survival rates than the WT mice (*P* = 0.0301) (Additional file 3: Figure S3). Taken together, these data suggest that γδ T cell deficiency protects *S. japonicum*-infected mice from liver fibrosis and damage caused by *Schistosoma* eggs.

### Reduced fibrosis is associated with decreased TGF-β and IL-17A in infected TCR δ KO mice

**Fig. 4:**
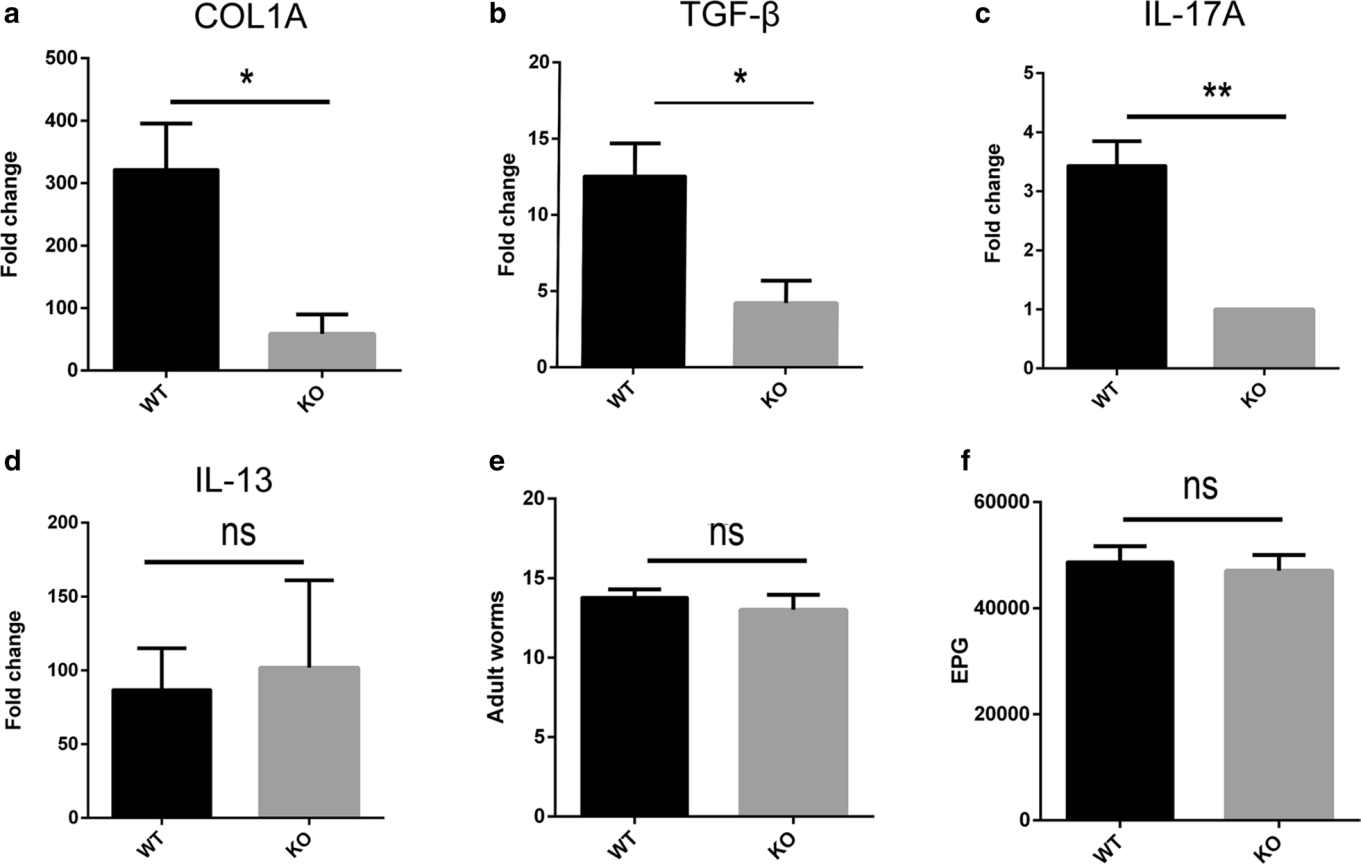
The fibrogenic genes were downregulated in *S. japonicum*–infected TCR δ KO mice. **a**-**d** Real-time RT-PCR measurement of mRNA transcripts of collagen I (**a**), TGF-β (**b**), IL-17A (**c**), and IL-13 (**d**) in WT and KO mice at 6 weeks post-infection. **e** Adult worms in WT and KO mice at 6 weeks post-infection. **f** Hepatic egg burden in WT and KO mice at 6 weeks post-infection. Differences were analyzed by using Student’s t-test, *n* = 3 or *n* = 5 per group, *P < 0.05, **P < 0.01. *Abbreviations*: ns, no significant difference; EPG, egg per gram

To study which factor is related to reduced fibrosis in *S. japonicum*-infected TCR δ KO mice, we extracted RNA from the liver tissue of the WT and KO mice at 6 weeks post-infection, and measured the mRNA transcripts of collagen, TGF-β, IL-17A and IL-13 using qRT-PCR. TCR δ deletion led to reduced expression of collagen I, TGF-β and IL-17A (*t*_(4)_ = 3.296, *P* = 0.0301, *t*_(4)_ = 3.218, *P* = 0.0324 and *t*_(4)_ = 5.966, *P* = 0.004, respectively) (Fig. 4a-c), but not IL-13 (Fig. 4d). To rule out the difference between the number of *Schistosoma* adults and the *Schistosoma* egg burden, we examined the total number of adults (Fig. 4e) and egg burden (Fig. 4f) in each mouse at 6 weeks post-infection. There was no significant difference between the findings for the WT and KO mice. The involvement of IL-17A and TGF-β was implicated in the fibrotic effects in the *S. japonicum* infection [28, 38, 39]. Taken together, our results indicate the involvement of TGF-β in alleviating the fibrosis in TCR δ KO mice infected with *S. japonicum*.

### The profibrotic effects of γδ T cells were associated with CD11b^+^Gr-1^+^ cells

**Fig. 5:**
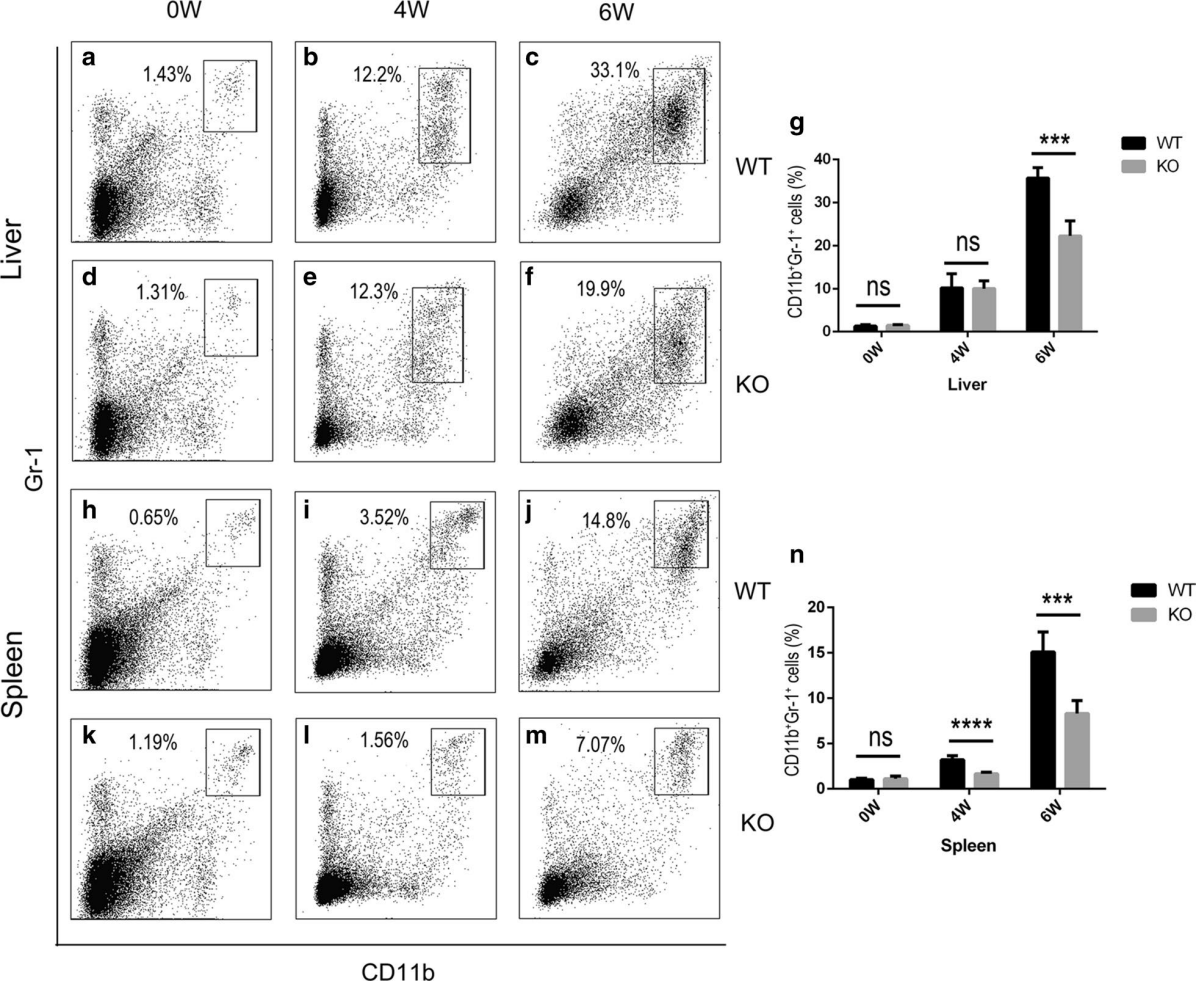
The profibrogenic effects of γδ T cells in infected mice are associated with CD11b^+^Gr-1^+^ cells. **a**-**c** The percentage of CD11b^+^Gr-1^+^ cells in WT mouse liver at 0 (**a**), 4 (**b**) and 6 (**c**) weeks post-infection, and representative dot plots. **d**-**f** The percentage of CD11b^+^Gr-1^+^ cells in KO mouse liver leukocytes at 0 (**d**), 4 (**e**) and 6 (**f**) weeks post-infection, and representative dot plots. **g** Statistical analysis of the percentage of CD11b^+^Gr-1^+^ cells in WT and mouse liver leukocytes. **h**-**j** The percentage of CD11b^+^Gr-1^+^ cells in WT mouse spleen at 0 (**h**), 4 (**i**) and 6 (**j**) weeks post-infection, and representative dot plots. **k**-**m** The percentage of CD11b^+^Gr-1^+^ cells in KO mouse spleen at 0 (**k**), 4 (**l**) and 6 (**m**) weeks post-infection, and representative dot plots. **n** Statistical analysis of the percentage of CD11b^+^Gr-1^+^ cells in WT and KO mouse spleen. Differences were analyzed by using Student’s t-test, *n* = 5 per group, ***P < 0.001; ns, no significant difference

In the WT mice, CD11b^+^Gr-1^+^ cells increased rapidly after infection (data not shown). To explore the relationship between γδ T cells and CD11b^+^Gr-1^+^ cells, we examined the percentage of CD11b^+^Gr-1^+^ cells in the liver and spleen from the WT and TCR δ KO mice at 0, 4 and 6 weeks post-infection. At 0 weeks post-infection, the WT and KO mouse livers had only about 1.4% and 1.3% CD11b^+^Gr-1^+^ cells, respectively (Fig. 5a, d), and there was no significant difference between the two groups (Fig. 5g). At 4 weeks post-infection, the percentage of CD11b^+^Gr-1^+^ cells in the WT and KO mouse livers increased to about 10.9% and 11.2%, respectively (Fig. 5b, e). At 6 weeks post-infection, the WT mouse livers had about 33% CD11b^+^Gr-1^+^ cells (Fig. 5c), while that in the KO mouse livers was about 20% (Fig. 5f), representing a significant reduction (*t*_(8)_ = 7.028, *P* = 0.0001) (Fig. 5g). At 0 weeks post-infection, the WT and KO mouse spleens had only about 0.6% and 1.2% CD11b^+^Gr-1^+^ cells, respectively (Fig. 5h, k); there was no significant difference between the two groups (Fig. 5n). At 4 weeks post-infection, the percentage CD11b^+^Gr-1^+^ cells in the WT mouse spleens increased to about 3.5% (Fig. 5i), and that in the KO mice was about 1.6% (Fig. 5l); there was a significant difference between the two groups (*t*_(8)_ = 7.212, *P* < 0.0001) (Fig. 5n). At 6 weeks post-infection, the percentage of CD11b^+^Gr-1^+^ cells in the WT mouse spleens continued to increase to around 15% (Fig. 5j). The percentage of CD11b^+^Gr-1^+^ cells in the KO mouse spleens decreased to around 7% (Fig. 5m), which was significantly lower than of wild-type mice (*t*_(8)_ = 5.748, *P* = 0.0004) (Fig. 5n). These data suggest that the fibrotic effects of γδ T cells in *S. japonicum* infection may be related to CD11b^+^Gr-1^+^ cells.

### The possible mechanisms of γδ cell promotion of liver fibrosis

**Fig. 6:**
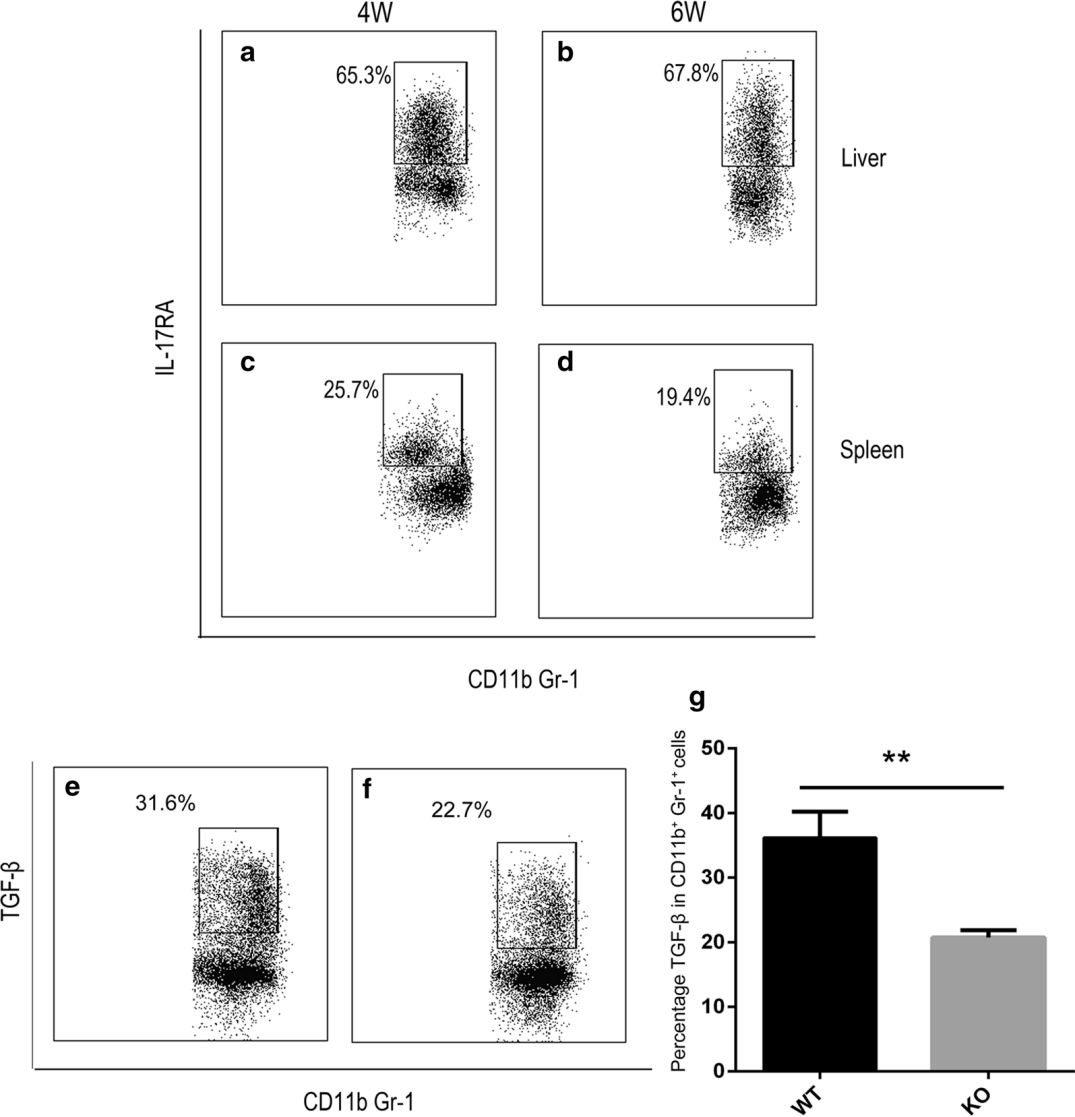
The possible mechanisms of γδ cell promotion of liver fibrosis. **a**, **b** CD11b^+^Gr-1^+^ cells in WT mouse liver expressed IL-17RA at 4 (**a**) and 6 (**b**) weeks post-infection. **c**, **d** CD11b^+^Gr-1^+^ cells in WT mouse spleen expressed IL-17RA at 4 (**c**) and 6 (**d**) weeks post-infection. **e** CD11b^+^Gr-1^+^ cells in WT mouse liver expressed TGF-β at 6 weeks post-infection. **f** CD11b^+^Gr-1^+^ cells in KO mouse liver expressed TGF-β at 6 weeks post-infection. **g** Statistical analysis of the percentage of TGF-β in CD11b^+^Gr-1^+^ cells from WT and KO mouse liver. Liver leukocytes were stimulated with PMA + ionomycin for 4 h; intracellular TGF-β staining was performed, and representative dot plots are shown. Student’s t-test was applied to two groups at six weeks, *n* = 5 per group, **P < 0.01

We interrogated the mechanism underlying the contribution of CD11b^+^Gr-1^+^ cells in mediating the fibrotic effects of γδ T cells. First, we examined the relationship between γδ cells and CD11b^+^Gr-1^+^ cells. Flow cytometric analysis showed that liver CD11b^+^Gr-1^+^ cells also expressed IL-17RA at 4 and 6 weeks post-infection (Fig. 6a, b). The same result was found for the spleen CD11b^+^Gr-1^+^ cells (Fig. 6c, d). As a receptor of IL-17A, IL-17RA may promote CD11b^+^Gr-1^+^ cell recruitment from the liver and spleen. In studying how CD11b^+^Gr-1^+^ cells promote fibrosis, we observed that the liver CD11b^+^Gr-1^+^ cells (WT and KO mice, about 35% and 20%, respectively) expressed TGF-β (Fig. 6e, f) (Additional file 2: Figure S2c shows the isotype). There was a significant difference between the two groups (*t*_(8)_ = 3.974, *P* = 0.0041) (Fig. 6g). These results suggest that IL-17A secreted by γδ T cells may directly contribute to the recruitment of CD11b^+^Gr-1^+^ cells and that CD11b^+^Gr-1^+^ cells may be associated with liver fibrosis *via* the expression of TGF-β.

## Discussion

Although the formation of granuloma and fibrosis limits the viability of *Schistosoma* eggs to a certain extent and helps improve the host survival rate, but the subsequent liver pathology is nonetheless very severe, and even leads to death. Therefore, more rational treatment of schistosomiasis requires more comprehensive understanding of granuloma and fibrosis formation. In the present study, we explored whether γδ T cells play a role in granuloma formation in the early stages of *S. japonicum* infection. Our results indicate that IL-17A-producing γδ T cells are involved in the processes of granuloma and fibrosis, although the abundance of the cells was relatively low. Moreover, the IL-17A-producing γδ T cells promoted the increase in CD11b^+^Gr-1^+^ cells. Further, we explored how γδ T cells promote CD11b^+^Gr-1^+^ cells. Our results suggest that γδ T cells may secrete IL-17A and mediate CD11b^+^Gr-1^+^ cell recruitment by engaging IL-17RA expressed on the CD11b^+^Gr-1^+^ cell surface. Next, we explored the possible mechanisms of fibrosis by CD11b^+^Gr-1^+^ cells. Our findings show that CD11b^+^Gr-1^+^ cells contribute to fibrogenesis by the high production of TGF-β.

γδ T cells are typically found at surface of barrier tissue such as the mucosa, lungs and skin. As innate T cells, γδ T cells are non-abundant; however, they play an important role in diverse diseases. For example, TCR-deficient mice are more susceptible to infection by bacteria and viruses, and have significantly increased incidence of tumors. γδ cells are well-documented as being involved in liver disease, such as non-alcoholic fatty liver disease, autoimmune hepatitis, liver fibrosis and cirrhosis and liver cancer [40–43]. Our results indicate that γδ T cells constitute approximately 3% of the total CD3^+^ T cells, and consist of two major subsets, which is consistent with previous reports [37, 44, 45]. In some disease models, Vγ1 cells, which are IFN-γ-producing γδ T cells, play an essential role in liver infection and liver cancer [46, 47]. In the present study, IFN-γ secretion by Vγ1 cells began to increase at 0 and 4 weeks post-infection, but then decreased at 6 weeks post-infection. Vγ2 cells, whose most striking feature is the secretion of IL-17A, are involved in immune responses and various diseases. They not only play protective roles against colitis and viruses, but also promote liver fibrosis [16, 48], especially, γδ T cells could exacerbate liver fibrosis by aiding the activation of HSCs in liver fibrosis induced by carbon tetrachloride injection [49]. Our observations show that during infection, the increase in Vγ2 cells was accompanied by an increase in their secreted IL-17A. These results suggest that IL-17A-producing γδ T cells (i.e. Vγ2 cells) may participate in liver pathological processes in mice infected with *S. japonicum.*

In the present study, we used TCR δ KO mice to confirm that γδ T cells are involved in liver fibrosis in *S. japonicum-*infected mice. Our results show that the KO mice had reduced granuloma area and attenuated liver fibrosis as compared with the WT mice. We selected granuloma caused by single eggs to ensure that the pathological improvement was due to the host immune response rather than the number of eggs, and this is consistent with previous reports [24, 50]. The improvement of liver function in the KO mice may have enhanced their survival rate as compared with the WT mice.

TGF-β and IL-13 are well-documented as two important cytokines of liver fibrosis in mice infected with *S. mansoni* and *S. japonicum*, and IL-13 is the key driving liver fibrosis [38, 39, 51, 52]. Here, TGF-β, and not IL-13, was decreased in the KO mouse livers at 6 weeks post-infection; IL-17A was also decreased in the KO mouse livers. These data suggest that the ameliorated liver fibrosis caused by the lower TGF-β was accompanied by decreased IL-17A expression.

CD11b^+^Gr-1^+^ cells are involved in diverse disease such as IBD, tumor, and liver fibrosis [53–55]. CD11b^+^Gr-1^+^ cells and γδ T cells are reciprocally regulated [16, 56]. Our data show that CD11b^+^Gr-1^+^ cells in the WT mice increased rapidly during infection. However, CD11b^+^Gr-1^+^ cells in the KO mice were decreased in comparison. These data indicate that γδ T cells may promote liver fibrosis by recruiting CD11b^+^Gr-1^+^ cells. The notable characteristic of γδ T cells is the expression of IL-17A, and our data show that CD11b^+^Gr-1^+^ cells can express IL-17RA. IL-17RA or the IL-17RA/IL-17RC heterodimer is considered the receptor of IL-17A [57], so we speculate that γδ T cells promote CD11b^+^Gr-1^+^ cells *via* an IL-17A–IL-17RA pathway. Compared with WT mice, liver fibrosis is decreased in IL-17RA-deficient mice [14]. In addition, IL-17RA deficiency can prevent the development of tubulointerstitial fibrosis in the kidney [58]. In two different models of airway fibrosis, IL-17RA-deficient mice were protected against both airway inflammation and fibrosis [59]. Finally, our data imply that CD11b^+^Gr-1^+^ cells facilitate the development of liver fibrosis by expressing TGF-β. Similarly, CD11b^+^Gr-1^+^ cells can promote silica-induced lung fibrosis in mice [60]. Taken together, we speculate that IL-17A may recruit CD11b^+^Gr-1^+^ cells and promote liver fibrosis by expressing TGF-β. However, we should point out that more studies and evidence are needed to reveal and clarify the integrated mechanism involved.

## Conclusions

We assessed the role of γδ T cells in liver fibrosis caused by acute murine schistosomiasis. We confirm that γδ T cells are involved in the development of liver fibrosis.

## Supplementary information

**Additional file 1: Figure S1.** Gating strategy for γδ T cells.

**Additional file 2: Figure S2.** Isotype controls for intracellular staining. **a**, **b** Isotype controls (BV421-labeled IgG2a) for IFN-γ (**a**) and IL-17A (**b**). **c** Isotype controls (PE-labeled IgG2a) for TGF-β.

**Additional file 3: Figure S3.** Survival curve of WT and TCR δ KO mice. The survival of two groups was monitored weekly for up to 16 weeks post-infection (*n* = 15 per group for one experiment).

## Data Availability

The datasets supporting the conclusions of this article are included within the article and its additional files.

## Abbreviations

DC: dendritic cell
Tfh cells: T follicular helper cells
Treg: regulatory T cells
MHC: major histocompatibility complex
ALT: aspartate aminotransferase
AST: aspartate aminotransferase, TGF-β: transforming growth factor β
IFN-γ: interferon-gamma
IL-13: interleukin-13
IL-17A: interleukin-17A
COL 1A: collagen 1A
