# Supplementary figures and images for: IL-17A-producing γδ T cells promote liver pathology in acute murine schistosomiasis
Source: Parasit Vectors. 2020 Jul 1;13:334. doi: 10.1186/s13071-020-04200-4 (PMC7329544; doi:10.1186/s13071-020-04200-4)

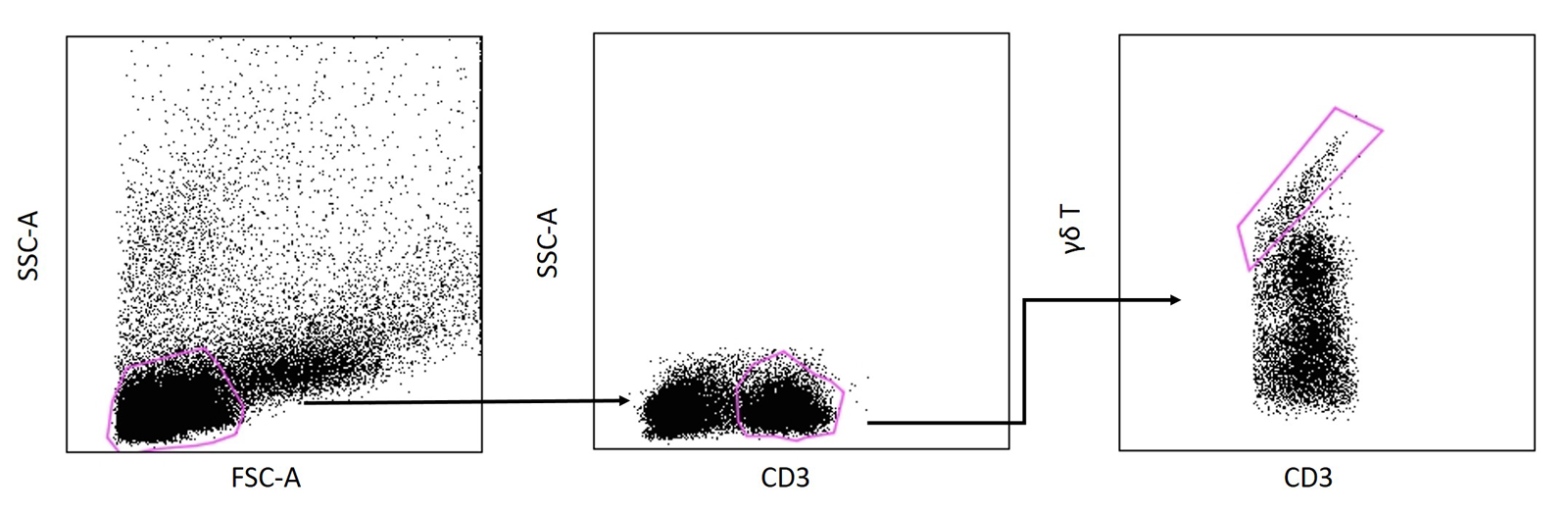

Supplement: Supplementary file 1 — Additional file 1: Figure S1. Gating strategy for γδ T cells. [file 13071_2020_4200_MOESM1_ESM.tif]

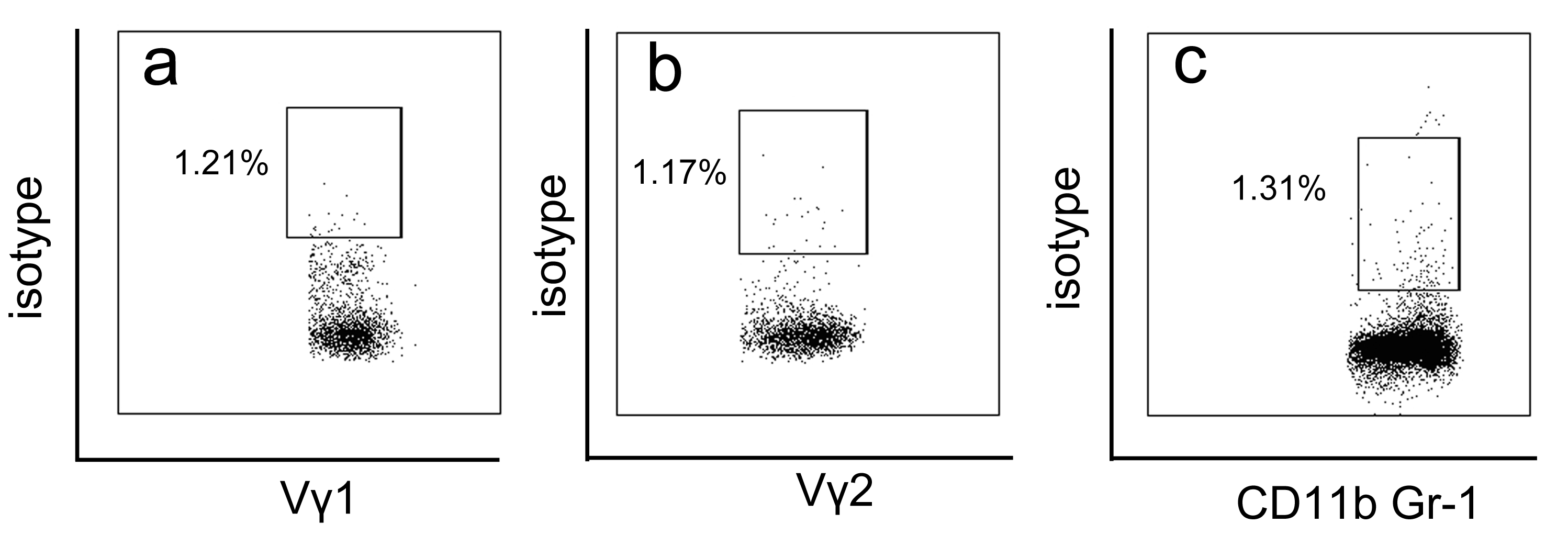

Supplement: Supplementary file 2 — Additional file 2: Figure S2. Isotype controls for intracellular staining. a, b Isotype controls (BV421-labeled IgG2a) for IFN-γ (a) and IL-17A (b). c Isotype controls (PE-labeled IgG2a) for TGF-β. [file 13071_2020_4200_MOESM2_ESM.tif]
